# Visual analysis of quality management in Chinese drug clinical trials based on CiteSpace and COOC

**DOI:** 10.3389/fmed.2025.1600915

**Published:** 2025-09-11

**Authors:** Jiayan Xia, Xuemei Ning, Lamei Jiang, Peipei Li, Jie Huang, Wenxiu Jiang, Wen Li, Cheng Wang, Linsha Zheng, Ting Jiang

**Affiliations:** ^1^The First Affiliated Hospital of Chengdu Medical College, Clinical Medical College, Chengdu Medical College, Chengdu, China; ^2^School of Great Health and Intelligent Engineering, Chengdu Medical College, Chengdu, China

**Keywords:** China, drug clinical trial, quality management, collaborative networks, evolutionary trends, visual analysis

## Abstract

**Background:**

Recently, considerable progress has been made in the quality of clinical trials conducted in China. However, the number of clinical trials conducted in China still falls below the global average standard. This study aims to identify research hotspots, collaborative networks, and evolutionary trends in the field of clinical trial quality management (CTQM) in China through bibliometrics and visual analyses to provide theoretical support and practical references for the optimization of domestipolicies.

**Methods:**

A systematic literature search was performed across the CNKI, Wanfang, and VIP databases to clinical trial quality management CTQM-related publications. Bibliometric analysis was conducted using CiteSpace 6.1.R6 and Co-Occurrence 20.5 (COOC 20.5), with key metrics including: annual output, active institutions, core journals, main authors, keywords, and thematic evolution. To capture internationally published works, supplementary searches were executed in Scopus, Web of Science, and PubMed for CTQM publications authored by Chinese scholars. Owing to the limited number of results (6 records), these documents were only included only in the discussion analysis.

**Results:**

A total of 528 articles were retrieved from the field of CTQM. The research process was divided into three periods: the basic standardization period (2003–2012), technology convergence period (2013–2019), and the intelligent transformation period (2020–2024). The theme shifted from the localization of the system to risk management, data management, and ethical governance driven by emerging technologies. The issuing organizations are primarily national-level administrative bodies, showing strong political-academic collaboration but limited cross-system partnerships. Artificial intelligence (AI)-based clinical trial quality management enhances quality control (QC) efficiency; however, it raises concerns about data privacy and ethical disparities.

**Conclusion:**

China’s research in the field of CTQM has led to the innovative integration of traditional quality control methods with new technologies. However, insufficient interdisciplinary cooperation and the absence of a data governance system pose ongoing challenges. In the future, it is necessary to build a three-dimensional ecosystem of “policy guidance, technological breakthroughs, and ethical synergy” to promote the rapid development of drug research in China.

## 1 Introduction

As a core stage in the development of new drugs, the quality management of clinical drug trials directly impacts the reliability of research data, the safety of trial participants, and the clinical value of approved drugs. With global investment in drug development continuing to grow, clinical trial quality management (CTQM) has become a key factor in ensuring data integrity, participant safety, and regulatory efficiency during the drug approval process (1, 2). Since China joined the International Council for Harmonization (ICH) in 2017 (3), CTQM practices have gradually aligned with international standards. To clarify the research priorities and development trends in China’s CTQM field, this study employed bibliometric tools such as CiteSpace 6.1.R6 and COOC 20.5 to conduct a multidimensional visualization analysis of CTQM-related literature. This analysis aims to reveal the annual output, active institutions, core journals, main authors, keywords, and thematic evolution in China’s CTQM field, providing evidence-based reference for the formulation of clinical trial quality management policies and the implementation and regulation of multinational clinical trials.

## 2 Materials and methods

### 2.1 Data sources

A systematic literature search was performed across the CNKI, Wanfang, and VIP databases to identify clinical trial quality management CTQM-related publications. The search string applied was: (“drug clinical trial” OR “pharmaceutical clinical trial” OR “good clinical practice”) AND (“quality control” OR “quality management” OR “quality assurance” OR “Risk Management”). A total of 1,333 articles were retrieved, with the following distribution: 280 from CNKI, 520 from Wanfang Data, and 533 from VIP Information.

To capture internationally published works, supplementary searches were executed in Scopus, Web of Science (WOS), and PubMed for CTQM publications authored by Chinese scholars. The three database search terms used were as follows: (1) TITLE-ABS-KEY [(“drug clinical trial” OR “pharmaceutical clinical trial” OR “good clinical practice”) AND (“quality control” OR “quality management” OR “quality assurance” OR “Risk Management”)] AND [LIMIT-TO (AFFILCOUNTRY, “China”)]; (2) {[“drug clinical trial”(Mesh) OR “pharmaceutical clinical trial” OR “Good Clinical Practice”(All Fields)] AND [“quality Assurance”(Mesh) OR “quality Control”(Mesh) OR “Risk Management”(Mesh) OR “Quality Management”(All Fields) OR “risk based monitoring”(All Fields)]}; (3) TS = [(“drug clinical trial” OR “pharmaceutical clinical trial” OR “good clinical practice”) AND (“quality control” OR “quality management” OR “quality assurance” OR “risk management”)]. This search yielded 264 articles published by Chinese authors. However, after two researchers (XN and LJ) reviewed them individually, only six were found to be relevant to the research topic. Given the small number of articles and their limited impact on the quantitative results of this study, we only included only these six articles in the discussion to ensure the completeness of our findings.

### 2.2 Inclusion and exclusion criteria

The inclusion criteria were as follows: (1) Study types: empirical research, systematic reviews, or policy analyses; (2) The title/abstract contains a clinical trial or drug clinical trial and at least one CTQM core term from the predefined lists: monitoring, data management, ethics review, risk management, quality control, auditing, protocol deviations and good clinical practice (GCP). (3) ≥ 50% of the results/discussion sections address CTQM processes (e.g., QC procedures for electronic data capture and risk-based monitoring workflows). (4) Literature form the establishment of the database to 30 December 2024. The following documents were excluded: (1) Duplicate publications and studies with incomplete information. (2) other types of publications (such as meeting abstracts, editorial materials, letters and early access, etc.).

### 2.3 Data processing

**FIGURE 1:**
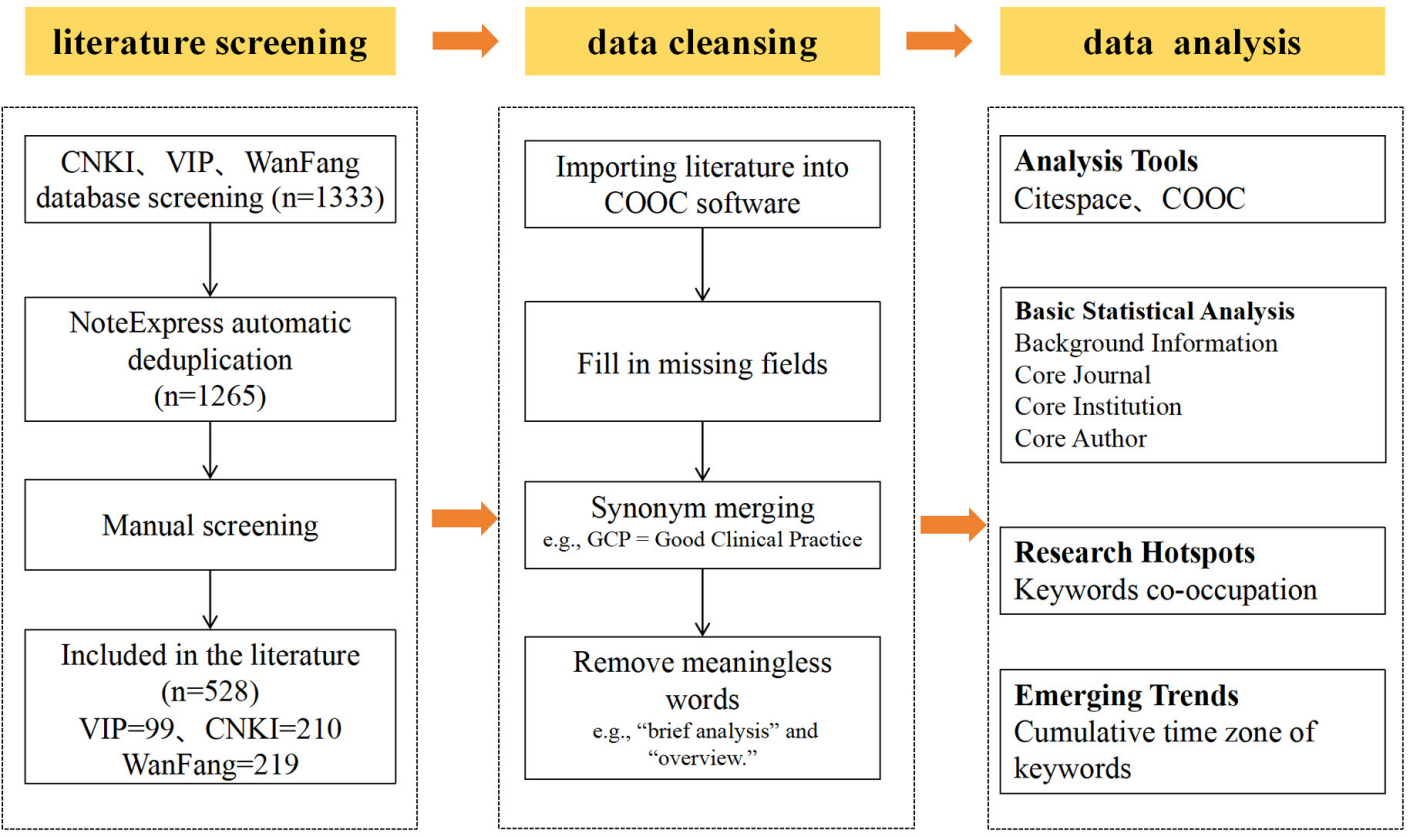
Flow chart of the research process.

Figure 1 presents the data processing and analysis. The literature obtained from each database was imported into NoteExpress for deduplication. Two researchers (XN and LJ) independently reviewed the literature; in cases of disagreement regarding inclusion, a third researcher was consulted to resolve the differences and reach a consensus. Of the literature obtained, 210 articles were retained from CNKI, 219 from Wanfang Data and 99 from VIP Information for analysis. The filtered literature was then imported into COOC 20.5 for further data cleaning. This included supplementing missing fields (e.g., keywords, institutions and authors), batch merging synonyms and deleting meaningless terms. COOC 20.5 was then used for the following analyses: publication statistics and frequency analysis: collaborative network analysis of institutions, authors and journals, and thematic evolution analysis based on keywords. CiteSpace 6.1.R6 was used to conduct keyword co-occurrence analysis, keyword clustering analysis and keyword burst detection. The parameters were set as follows: Time Slicing (Year per Slice) = 1 year; TopN = 50.

## 3 Results and analysis

### 3.1 Overview of publication trends

**FIGURE 2:**
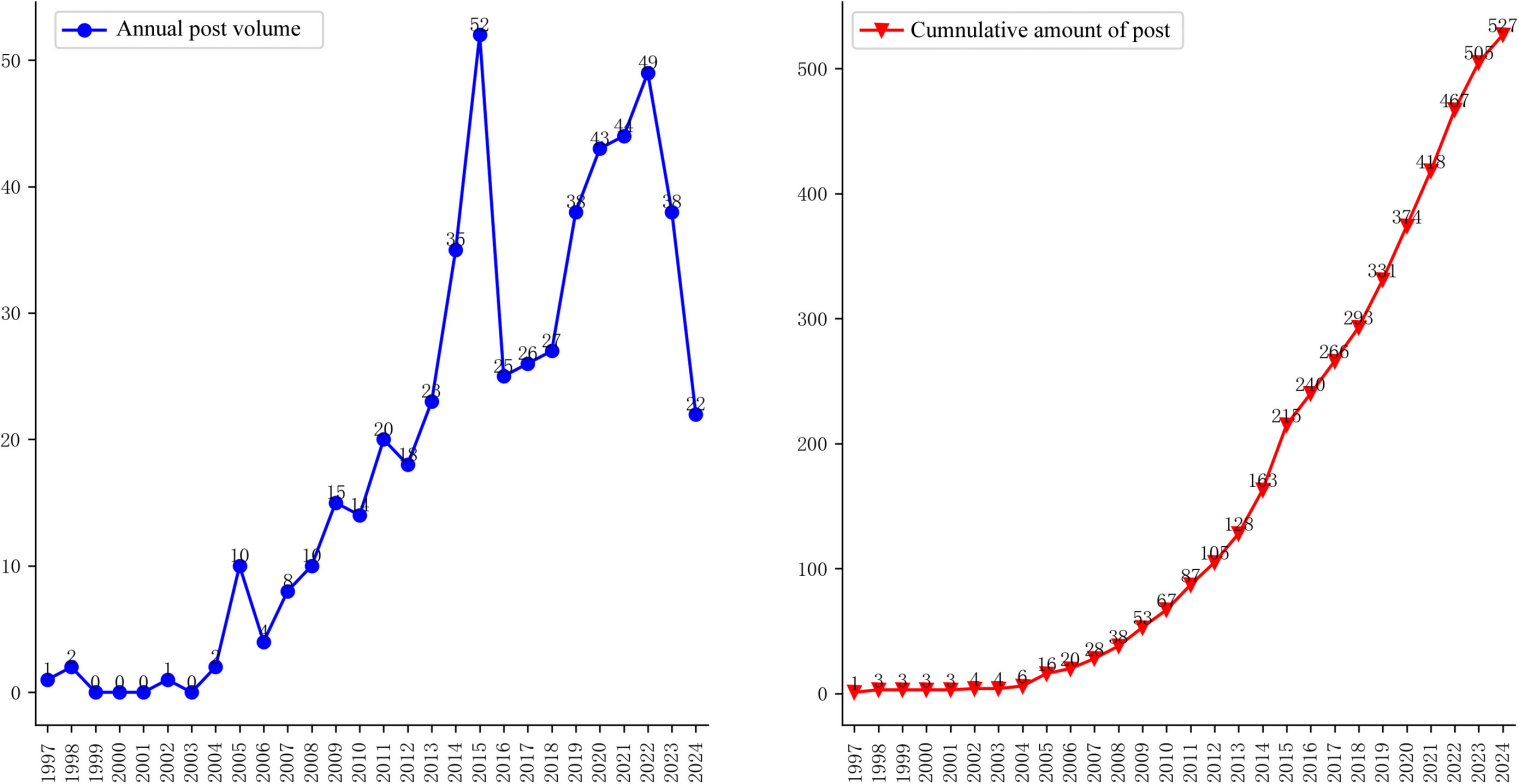
Annual and cumulative number of articles issued.

Figure 2 illustrates the annual and cumulative publication trends in the field of CTQM between 1997 and 2024. The results demonstrate that Chinese research originated from Yao’s (4) pioneering work in 1997, when his team first systematically proposed the idea of achieving scientific and objective evaluation of clinical efficacy through the implementation of internationally standardized GCP. This laid the theoretical foundation for subsequent studies.

From a temporal evolution perspective (Figure 2), research has progressed in three distinct phases. Emergence phase (1997–2003): annual publications averaging fewer than 1, with research themes concentrated on foundational concept introduction and policy framework exploration (5–8). Slow development phase (2004–2010): The number of annual publications increased to 9. Rapid growth phase (2011–2024): Annual publications surged to 33.8, with dual peaks observed in 2015 (52 publications) and 2022 (49 publications), during which research hotspots became prominently concentrated.

**FIGURE 3:**
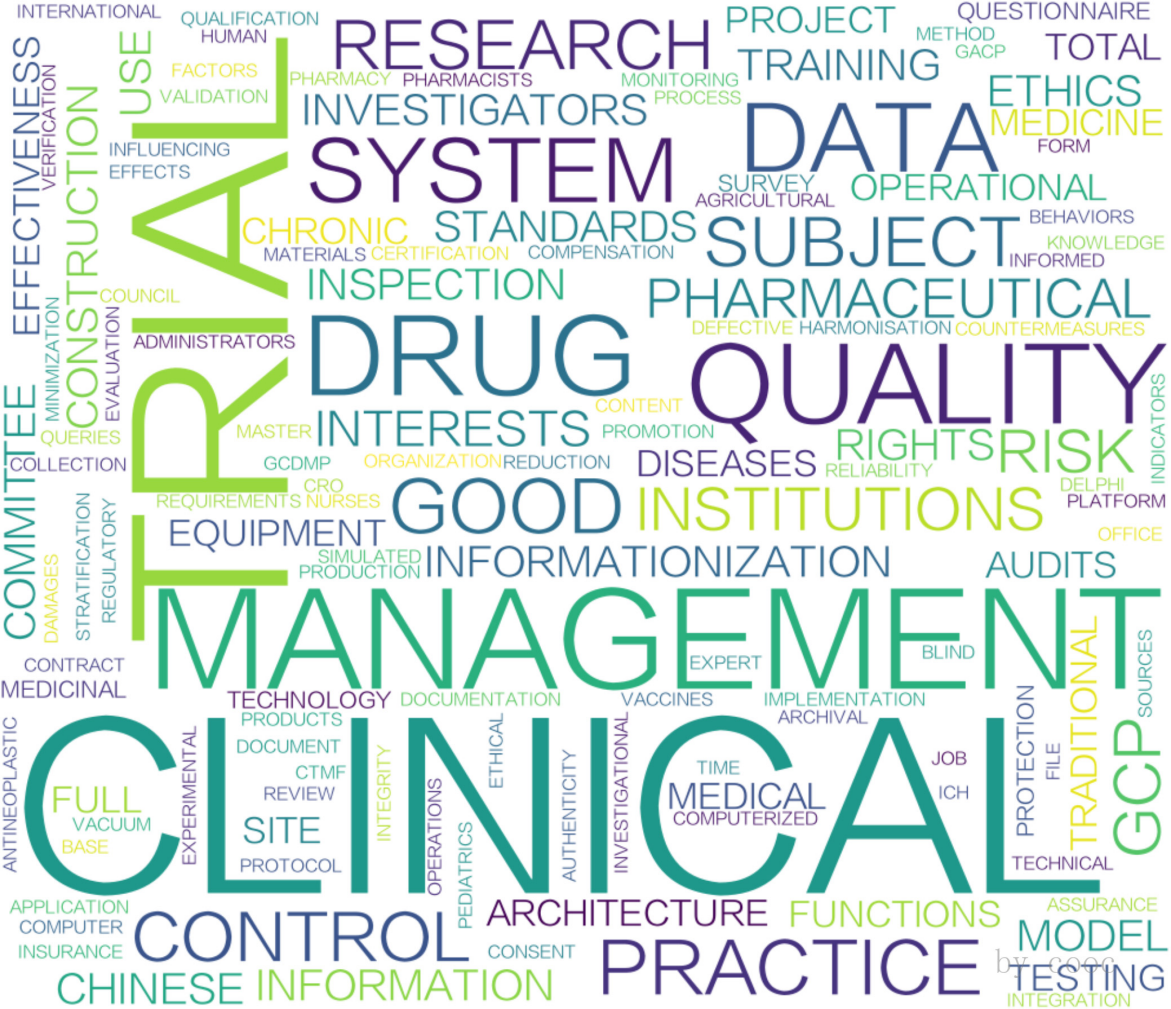
Word cloud of keywords for 2015 issuance.

**FIGURE 4:**
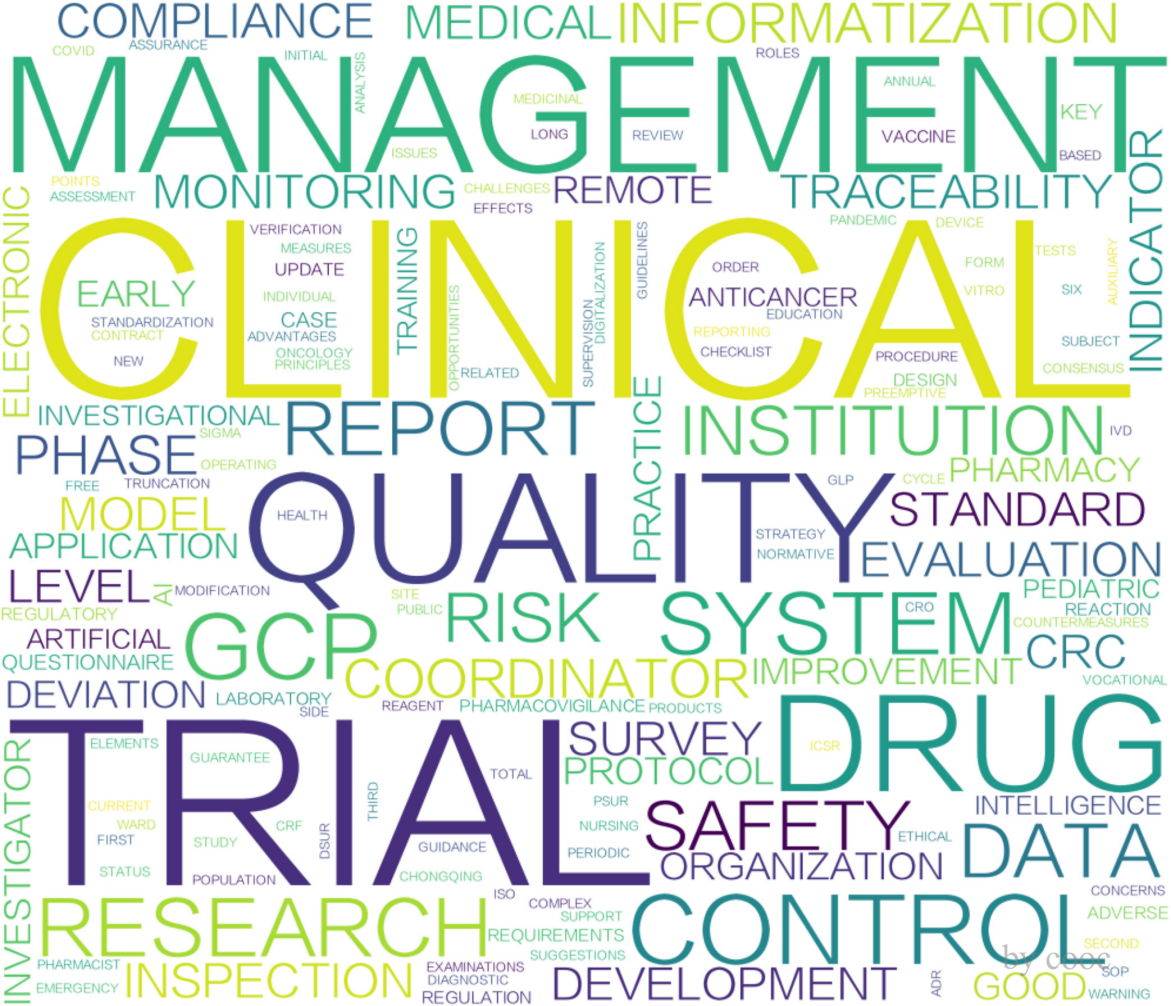
Word cloud of keywords for 2022 issuance.

According to the analysis shown in Figures 3, 4, the two peaks are both concentrated in the areas of clinical trial quality management, risk management, information management and clinical trial institutions. Additionally, research on data management and pharmaceutical regulation was highlighted in 2015 (Figure 3), whereas studies on artificial intelligence and protocol deviations rose in popularity in 2022 (Figure 4). Remarkably, following China’s 2017 accession to the ICH, publication output rose at an average annual rate of 24% (2017–2024). While this temporal association suggests that international alignment policies may have contributed to the increase, we cannot rule out the influence of other concurrent factors.

### 3.2 Analysis of publishing institution characteristics

#### 3.2.1 Core institution distribution

This study examined 528 articles from 250 institutions. An analysis of the top 10 issuing organizations (Table 1), revealed that the Shanghai Center for Drug Evaluation and Inspection led with 10 publications, followed by the center for Drug Evaluation and the Center for Drug Certification under the National Medical Products Administration (NMPA), each contributing 7 publications. Strikingly, the top three are government agencies, reflecting a strong emphasis on drug quality control within China’s regulatory system. In addition, hospitals and universities are also important publishing institutions.

**TABLE 1:** Analysis of the top 10 issuing organizations.

No.	Institution	Number of publications	Institution type
#1	Shanghai Center for Drug Evaluation and Inspection	10	Government agency
#2	Center for Drug Evaluation, State Food and Drug Administration	7	Government agency (directly under the State Council)
#3	Center for Certification of Drugs, State Food and Drug Administration	7	Government agency (directly under the State Council)
#4	Shanghai University of Traditional Chinese Medicine Affiliated Shuguang Hospital	6	Hospital
#5	Shenyang Pharmaceutical University	6	University
#6	Beijing Hospital	5	Hospital
#7	Central South University, Xiangya School of Public Health	6	University
#8	Tianjin University of Traditional Chinese Medicine	5	University
#9	West China Hospital Affiliated with Sichuan University	5	University
#10	Hunan Cancer Hospital	5	Hospital

National institutions have various research focuses. The NMPA is focused primarily focused on policy implementation and full-cycle clinical trial supervision, such as internal audits, onsite inspections, risk management, and data security (9–12), it also draws on the Food and Drug Administration (FDA) and European Medicines Agency (EMA) regulatory cases to develop dynamic quality improvement strategies (13, 14). Moreover, the Shanghai Center for Drug Evaluation and Inspection emphasized innovative regulatory approaches, producing notable work on topics such as sponsor responsibility frameworks, decentralized trial management, and digital regulatory methods (15–18).

#### 3.2.2 Institutional collaboration network

**FIGURE 5:**
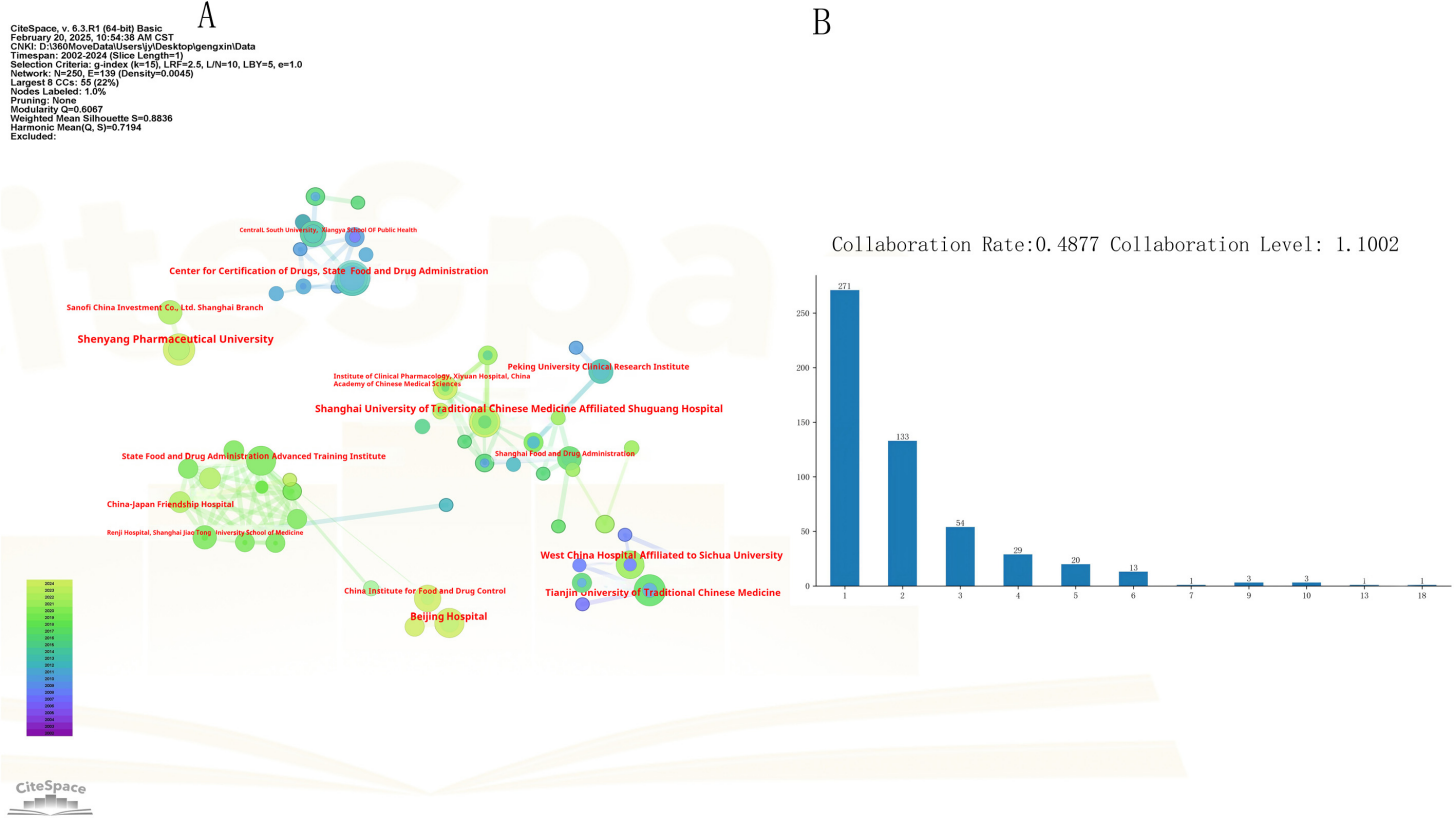
Institutional collaboration analysis. **(A)** Institutional collaboration; **(B)** collaboration degree.

The institutional collaboration network (2002–2024) constructed via CiteSpace exhibits significant clustering characteristics: network density = 0.017, modularity Q = 0.6 (> 0.3 threshold), and mean silhouette coefficient S = 0.88 (> 0.7 threshold), confirming robust clustering effects (Figure 5A). Figure 5B shows that the institutional collaboration rate is 0.48, with a collaboration level of 1.10. Of these collaborations, 23.6% occurred three or more times. This study identified three primary cross-sector collaboration models within China’s CTQM research ecosystem: (1) agency-university collaboration models, exemplified by the ongoing collaboration between the Drug Evaluation Center of the National Medical Products Administration and the School of Public Health at Central South University; (2) agency-hospital alliances, such as the collaborative partnership between the Shanghai Drug Administration and the Shanghai University of Traditional Chinese Medicine Affiliated Shuguang Hospital; (3) hospital-university networks, such as the established cooperation framework between West China Hospital of Sichuan University and Tianjin University of Traditional Chinese Medicine. In addition, hospitals and schools maintain strong internal industry connections, forming academic alliances (university-to-university collaboration) and hospital clusters (hospital-to-hospital collaboration).

### 3.3 Author collaboration network analysis

**FIGURE 6:**
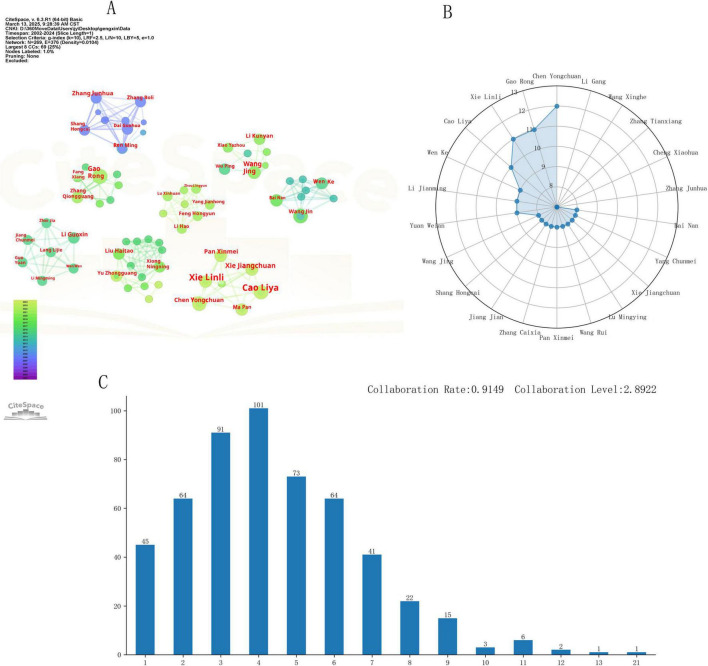
Statistics on authors of publications. **(A)** Core author radar chart; **(B)** author collaboration network; **(C)** author collaboration degree.

The author collaboration network (Figure 6A) shows that 272 nodes with 360 connecting lines formed a cooperative cluster with significant clustering characteristics (modularity Q = 0.59, mean profile coefficient S = 0.86). The author collaboration rate was 0.92, and the collaboration degree was 2.89 (Figure 6C). These findings indicate that the academic community exhibited a highly structured collaboration pattern.

Based on Price’s (19) Law, the threshold for core authors was calculated as M = 0.749$\sqrt{\text{Nmax}}\approx$ 3. The maximum number of papers published by an author among the 1,661 authors was 12. A total of 172 core authors (with ≥ 3 published papers) were identified, accounting for 10.36% of the total number of authors. Twenty-two highly productive scholars (with ≥ 7 papers published) formed the core research group (Figure 6B), such as Chen Yongchuan and Gao Rong, who typically collaborate closely with other authors (Figure 6A), indirectly indicating that strengthening collaboration can increase research output.

### 3.4 Issuing journals

A total of 528 articles were scattered across 127 journals. Sixteen titles contributed six or more papers, collectively accounting for 360 publications (68.2%). As shown in Table 2, Chinese New Drugs published the largest share (*n* = 74; IF = 1.908), followed by the Chinese Journal of Clinical Pharmacology (*n* = 58; IF = 1.851), Chinese New Drugs and Clinical Remedies (*n* = 53; IF = 1.529), and China Pharmacy (*n* = 39; IF = 2.414).

**TABLE 2:** Journal publication statistics.

Journal name	Number of publications
Chinese Journal of New Drugs	74
Chinese Journal of Clinical Pharmacology	58
Chinese Journal of New Drugs and Clinical Remedies	53
China Pharmacy	39
China Pharmaceuticals	20
Chinese Journal of Clinical Pharmacology and Therapeutics	17
Herald of Medicine and Pharmacy	17
Chinese Pharmaceutical Affairs	16
Chinese Journal of Hospital Pharmacy	13
Chinese Medical Ethics	10
China Pharmacist	9
Acta Pharmaceutica Sinica	8
Chinese Journal of Medical Science Research Management	7
Drug Evaluation Research	7
Journal of Pediatric Pharmacy	6
Shanghai Medical and Pharmaceutical Journal	6

### 3.5 Keyword burst analysis

Table 3 presents the frequency, betweenness centrality, and year of the first appearance of high-frequency keywords, revealing the development process of CTQM research. Betweenness centrality analysis revealed that clinical trials (0.62), drug clinical trials (0.50), quality control (0.25), good clinical practice (0.21), and drug clinical trial organizations (0.19) form the core framework of the CTQM knowledge network, establishing long-term connections between standard formulation and implementation research. In contrast, emerging themes such as risk management (0.05), data management (0.01), ethical review (0.02), and informatization (0.01), which emerged after 2015, exhibit low centrality, indicating that they remain on the periphery of the network and rely on traditional core terms for access. This suggests the need to establish stronger cross-domain bridges in the areas of risk, information, data, and ethics in the future.

**TABLE 3:** Analysis of high-frequency keywords.

Serial number	Byword	Frequency	Betweenness centrality (rb)	Year of first occurrence
#1	Clinical trial	196	0.62	2005
#2	Drug clinical trials	150	0.5	2005
#3	Quality control	118	0.25	2004
#4	Good Clinical Practice	93	0.21	2002
#5	Drug clinical trial organizations	47	0.19	2009
#6	Risk management	37	0.05	2015
#7	Ethical review	13	0.02	2020
#8	Total Quality Management	16	0.01	2008
#9	Data management	18	0.01	2018
#10	Informatization	17	0.01	2016

### 3.6 Keywords clustering analysis

Keywords clustering analysis based on the log-likelihood ratio (LLR) algorithm and mutual information (MI) metrics identified 11 valid clusters (modularity Q = 0.5139, average silhouette coefficient S = 0.8225) (Table 4). Six clusters presented S values > 0.7, indicating strong internal keyword associations and rational classification. In clustering, the higher the LLR or MI is, the better the metric reflets the theme content of the cluster.

**TABLE 4:** Keyword cluster analysis.

Cluster ID	Number of nodes	Contour value (S)	Starting year	Clustering label	LLR (Top 2)	MI (Top 2)
#0	48	0.701	2013	Drug clinical trials	Drug clinical trials (66.02)	Normative standards (1.17)
					Quality control (55.25)	Quality assurance system (1.17)
#1	38	0.898	2015	Clinical trial	Clinical trial (56.58)	Change of production site (1.07)
					Data management (23.1)	Regulatory model (1.07)
#2	24	0.749	2015	Good clinical practice	Data verification (13.11)	Information technology management (0.42)
					Apply for (10.34)	
#3	21	0.813	2015	Drug clinical trial organizations	Management model (11.97)	Quality management evaluation system (0.21)
					Clinical research coordinator (7.18)	
#4	29	0.7	2015	Quality control	Quality management (70.92)	Clinical trials of oncology drugs (0.45)
					Antitumor drugs (8.51)	
#5	10	0.918	2019	Risk management	Ethical review (14.04)	Compliance review (0.14)
					Hierarchical analysis (12.25)	

The drug clinical trial cluster (Cluster 0) was the largest cluster (48 nodes, S = 0.701), which originated in 2013 and focused on core themes such as “drug clinical trials” (LLR = 66.02), “quality control” (LLR = 55.25), and “normative standards” (MI = 1.17), and “quality assurance system” (MI = 1.17). This once again demonstrates the central role of basic standard setting and management systems in CTQM research. Cluster 1 includes “data management” (LLR = 23.1) and “change of production site” (MI = 1.07), whereas Cluster 2 includes “data verification” (LLR = 13.11) and “information technology management” (MI = 0.42). Both clusters are highly associated with data management and informatization technologies in CTQM. Cluster 3 aggregates research on drug clinical trial institutions, featuring “management model” (LLR = 11.97), “clinical research coordinator” (LLR = 7.18), and “quality management evaluation system” (MI = 0.21), and the research focus lies primarily on institutional operational models and personnel management. Cluster 4 (quality control) highlights “antitumor drugs” (LLR = 8.51). The risk-control theme (Cluster 5), formed in 2019, centers on ethical review (14.04).

### 3.7 Thematic evolution and hotspot analysis

**FIGURE 7:**
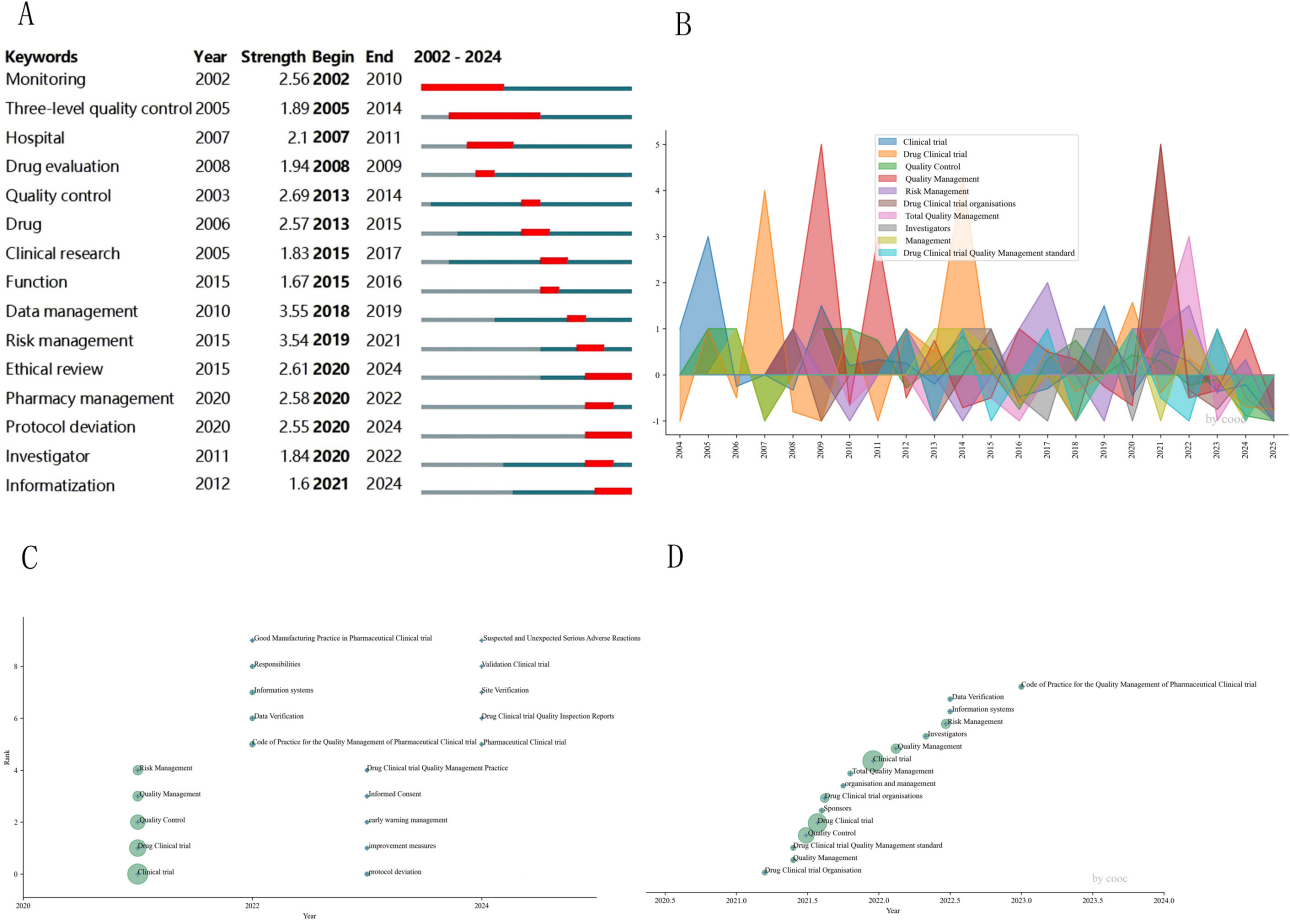
Keyword hotspot evolution analysis. **(A)** Keyword emergence visualization mapping; **(B)** annual growth rate; **(C)** Thematic evolution cumulative time-region chart (2020–2024); **(D)** weighted temporal evolution map of research themes (three-level quality control-level I: project group quality control, level II: professional group quality control, level III: institutional office quality control).

CiteSpace was used to generate a keyword-burst map (Figure 7A), In contrast, COOC produced a hotspot-shift diagram (Figure 7B) and thematic-evolution maps (Figures 7C, D) to explore the evolutionary trajectory and shifting hotspots of clinical-trial quality control in China.

The evolutionary trajectory of CTQM research delineates three sequential phases (Figure 7): 2003–2012 was dominated by foundational studies, as evidenced by active themes such as “drug clinical trials” and “quality control” (Figure 7B) and the emergence of burst keywords such as “regulation” (burst = 2.56), “hospital” (2.10), and “three-level quality control (20)” (1.89) (Figure 7A). From 2013 to 2019, the field shifted toward a technologization trajectory, with “risk management” reaching its peak (Figure 7B) and concurrent bursts of “drug” (2.57), “data management” (3.55), and “risk management” (3.54). From 2020 onward, the paradigm experienced an intelligent leap, as “informatization management,” “ethical review,” and “protocol deviation” rapidly ascended to the research frontier (Figures 7A, C, D).

## 4 Discussion

### 4.1 Stage leap in research paradigm

The results of this study show that China’s CTQM research followed a three-stage path of “basic specification→technology integration→intelligent transformation,” which is an evolutionary trajectory defined by both policy regulations and literature visualization.

(1)    Basic Standardization Period (2003–2012): Initiated with the enactment of China’s 2003 *Good Clinical Practice* (21), this phase witnessed concerted efforts by researchers to standardize and localize clinical trial implementation. The dominant themes included quality control, monitoring, and hospital management (Figure 7A), with Cluster 1 (“Drug Clinical Trials”) revealing strongly associated term pairs: standardized criteria (MI = 1.17) and quality assurance systems (MI = 1.17) (Table 4). These metrics collectively validate the foundational stage of CTQM research, characterized by systematic norm establishment and procedural codification.
(2)    Technology Convergence Period (2013–2019): The 2013 interpretation of “*Technical Guidance for Clinical Trial Data Management*” mandated nationwide adoption of electronic data capture (EDC) systems (22), marking China’s pivotal transition from paper-based/manual regulatory models toward digitalized, technology-driven oversight. This shift propelled data management and risk management into research prominence (Figure 7), with EDC utilization increasing from 12% (2012) to 61% (2016) (23). China’s accession to the ICH in 2017 further accelerated international technical convergence. Inspired by this integration, the 2018 NMPA *Standards and Procedures for Rapid Safety Data Reporting in Drug Clinical Trials* (24) established a robust framework for fusing risk surveillance with real-time reporting technologies.
(3)    Intelligent Transformation Period (Post-2020): The FDA’s approval in 2019 of the first AI-assisted auditing system marked a global inflection point in trial oversight (25). This was closely followed by China’s pivotal 2020 policy: the “Guiding Principles for Real-World Evidence Supporting Drug Development and Review (Trial),” issued by the NMPA (26). This policy formally integrated AI and blockchain technologies into the clinical trial regulatory framework, accelerating intelligent transformation across the industry. Bibliometric evidence confirms this shift, with digital governance exhibiting significant growth after 2021 (Figure 7) and emerging as a dominant research focus. Most recently, China’s release of the Pharmaceutical Industry Digital-Intelligent Transformation Implementation Plan (2025–2030) in April 2025 mandated a comprehensive digital and intelligent upgrade of the entire pharmaceutical value chain by 2030 (27), thus initiating an era of systematic intelligent governance.

### 4.2 Synergistic innovation mechanisms in core communities

An analysis of institutional and author collaboration networks (sections “3.2 Analysis of publishing institution characteristics” and “3.3 Author collaboration network analysis”) indicates that government-academia-hospital partnerships are the primary drivers of CTQM research in China. This collaborative framework has significantly increased research output, as evidenced by the positive correlation between collaboration levels and publication rates. These findings are consistent with the principle of promoting regulatory science through “government-industry-research” synergy, which is endorsed domestically (28).

However, compared with international standards, the scope of CTQM development in China is relatively insufficient in terms of cross-system collaboration. The subordinate agencies of the NMPA played a leading role in the early stages of CTQM development (Table 1). In subsequent stages, however, mechanisms for deeper integration with industry, academia and research institutions have remained inadequate and most collaborations have been limited to bilateral partnerships. In contrast, Europe and the United States emphasize establishing a multistakeholder collaboration network that includes sponsors (pharmaceutical companies), contract research organizations, academic medical centers and regulatory agencies (29). In order to advance CTQM research and enhance its global influence, China should prioritize expanding cross-system collaboration, strengthening deep cooperation with the industrial sector and actively integrating into global multicenter clinical trial networks.

### 4.3 Dual-edged effects of emerging technologies

Technologies such as artificial intelligence (LLR = 10.48) and blockchain (burst intensity = 7.5) have driven progress in smart monitoring and ethical governance. However, they have also exacerbated challenges such as data silos and privacy risks. Vallée (30) highlighted that, although digital twin technology has the potential to transform precision medicine and patient outcomes, it has also sparked significant controversy regarding data privacy and ethics. Harvey and Gowda (25) further elaborate on the complex regulatory balance that the FDA must maintain when regulating AI-based medical applications. Chinese research has recognized this global challenge, demonstrating foresight through preliminary exploration of ethical governance frameworks. In 2022, Liu et al. (31) from Central South University addressed the ethical challenges of AI-driven clinical trials by developing strategies to optimize performance while ensuring ethical integrity. Li and Yang (32) advocate resolving ethical conflicts through human-centered design to prevent technological alienation, and bridging the data divide through people-centered governance. Although China has achieved localized development in ethical governance, it still needs to address the global issue of “technological and ethical imbalance” by coordinating technological empowerment with regulatory frameworks. To address the widespread issue of “technology–ethics mismatch,” the international community must urgently establish comprehensive ethical standards and data governance mechanisms that are in line with the pace and scale of technological development.

### 4.4 Synergistic influence of international insights and China’s contributions

#### 4.4.1 Imperatives for leveraging international maturity in achieving China’s development goals

Advancing RBQM localization: The risk-based quality management (RBQM) framework endorsed by ICH E6(R2) (2) constitutes a globally recognized model. In light of the increasing domestic focus on risk management, China should further align RBQM principles with the unique characteristics of its clinical trial landscape, such as the variability in site capabilities and sponsor expertise. This alignment calls for the development of more pragmatic and flexible RBQM implementation guidelines and tools to increase the efficiency of resource allocation.

Driving the development of decentralized clinical trial (DCT) in China: Europe and the United States are at the forefront of DCT development, driving advancements in DCT methodology (33). The research hotspots identified in this study, namely “digitisation” and “digital regulation,” provide a strategic foundation for China to develop a DCT path that is tailored to its national circumstances, such as making use of mobile health technologies and remote monitoring systems. Furthermore, proactive strategies must be implemented to address the emerging challenges posed by DCT, particularly concerning data security and the safeguarding of participant rights.

Establishing an agile AI governance framework: Building upon regulatory precedents such as the FDA’s guidance (25) and the EU AI Act (34), China must accelerate the formulation of comprehensive evaluation criteria, validation protocols, and ethical oversight guidelines for the application of AI and other emerging technologies in CTQM. Such efforts are essential for fostering innovation while maintaining effective risk management.

#### 4.4.2 Global value proposition of China’s evolving experience

After searching domestic and international databases, it appears that no systematic bibliometric analysis has been conducted using CiteSpace, which specifically targets the CTQM subfield under China’s regulatory practice framework. This study summarizes the development, evolution, research frontiers, and research shortcomings of CTQM in China, demonstrating the unique value of China’s experience.

Transformation insights from governance experiences: China is currently in a critical phase of transitioning from a global regulatory follower to an innovator. This transformation is characterized by the rapid development of a scientific regulatory framework, the accelerated construction of infrastructure and the establishment of a national network of clinical trial sites (35–39). The unique challenges encountered during this period of rapid change, such as conducting large-scale trials (40) and ensuring traceability across the entire supply chain (41), have provided valuable governance insights. These experiences provide valuable reference material for other emerging pharmaceutical markets that are undergoing similar transformative phases.

Innovation under resource constraints: Operating in contexts marked by relative resource scarcity or uneven distribution, Chinese researchers have developed cost-effective quality control strategies (42, 43), investigated targeted technologies (e.g., AI applications) (44, 45), and formulated localized implementation models (46, 47) that enhance CTQM efficiency. These innovations provide globally relevant insights into sustaining high-quality clinical trial standards under constrained conditions.

Eastern ethical perspectives on emerging technologies: Ethical governance frameworks proposed by Chinese scholars centered on principles of “human–centricity” [to counteract technological alienation (44)—the risk that AI-driven decision-making deprioritizes human values and clinical judgment] and “public welfare” [to address data inequities (48)—systematic disparities in data access, quality and representativeness among regions or populations]. These contributions enrich the global discourse on creating more inclusive and humanistic ethical frameworks for intelligent technologies.

### 4.5 Limitations and future work

This study has several limitations. Not all foreign databases were covered, which may have resulted in incomplete literature inclusion. Additionally, the results of the study exhibit significant regional characteristics, which limits the replicability of policy systems. China’s CTQM mechanism, which is driven by the government, fundamentally differs from enterprise-driven models in Europe and the United States. Policy transplantation teams must be aware of the risks associated with contextual adaptation. When applied in other countries, it must be adapted to the unique characteristics of local systems.

We intend to conduct thorough research into the status of drug clinical trials worldwide, establish a “global drug clinical trial quality benchmarking system,” and integrate China’s drug clinical trials further with national policies. This will promote the integration of Chinese practices with international developments.

## 5 Conclusion

China’s research on CTQM has evolved by building an institutional foundation, technological empowerment, and intelligent transformation, forming an innovative “dual-track advancement” framework that integrates traditional quality control with emerging technologies. However, insufficient interdisciplinary collaboration and gaps in data governance remain key challenges. Future efforts should establish a tripartite ecosystem (policy guidance-technological breakthroughs-ethical synergy) for CTQM research in China, thereby contributing Chinese insights to the drug development process worldwide.

## Data Availability

The datasets presented in this study can be found in online repositories. The names of the repository/repositories and accession number(s) can be found in this article/Supplementary material.
